# *S*-Allylmercapro-*N*-Acetylcysteine Attenuates the Oxidation-Induced Lens Opacification and Retinal Pigment Epithelial Cell Death In Vitro

**DOI:** 10.3390/antiox8010025

**Published:** 2019-01-16

**Authors:** Naphtali Savion, Samia Dahamshi, Milana Morein, Shlomo Kotev-Emeth

**Affiliations:** Goldschleger Eye Research Institute and Department of Human Molecular Genetics and Biochemistry, Sackler Faculty of Medicine, Tel Aviv University, Tel-Aviv 61390, Israel; sdahamshi@gmail.com (S.D.); tormoz111@hotmail.com (M.M.); kotevs@tauex.tau.ac.il (S.K.-E.)

**Keywords:** oxidation, antioxidant, RPE, cataract, *S*-Allylmercapro-*N*-Acetylcysteine

## Abstract

The capacity of *S*-Allylmercapto-*N*-acetylcysteine (ASSNAC) to protect human retinal pigment epithelial (RPE) cells (line ARPE-19) and porcine lenses from oxidative stress was studied. Confluent ARPE-19 cultures were incubated with ASSNAC or *N*-acetyl-cysteine (NAC) followed by exposure to oxidants and glutathione level and cell survival were determined. Porcine lenses were incubated with ASSNAC and then exposed to H_2_O_2_ followed by lens opacity measurement and determination of glutathione (reduced (GSH) and oxidized (GSSG)) in isolated lens adhering epithelial cells (lens capsule) and fiber cells consisting the lens cortex and nucleus (lens core). In ARPE-19 cultures, ASSNAC (0.2 mM; 24 h) increased glutathione level by 2–2.5-fold with significantly higher increase in GSH compared to NAC treated cultures. Similarly, ex-vivo exposure of lenses to ASSNAC (1 mM) significantly reduced the GSSG level and prevented H_2_O_2_ (0.5 mM)-induced lens opacification. These results demonstrate that ASSNAC up-regulates glutathione level in RPE cells and protects them from oxidative stress-induced cell death as well as protects lenses from oxidative stress-induced opacity. Further validation of these results in animal models may suggest a potential use for ASSNAC as a protective therapy in retinal degenerative diseases as well as in attenuation of oxidative stress-induced lens opacity.

## 1. Introduction

Age-related macular degeneration (AMD) and cataract are leading causes of blindness among the elderly population in the world [1]. AMD appears in two clinical types, the neovascular wet type and the atrophic dry type. Retinal pigment epithelial (RPE) cells are located between the light-sensing photoreceptor cells and the chorio-capillaries and their oxidative stress-induced apoptosis is considered to be a crucial causal factor in photoreceptor cells death, resulting in the initiation and progression of atrophic dry type AMD [2,3,4]. 

Cataract presents as lens opacity, which was found to be associated with oxidative stress [5,6] and an elevated level of H_2_O_2_, suggesting that the H_2_O_2_-induced oxidative stress causes oxidation of lens proteins and lipids leading to lens opacification [7,8]. Furthermore, it has been shown that systemic hypertension significantly increases the risk of cataract formation in the rat eyes through depletion of cellular antioxidants such as glutathione and elevation of oxidized lipids as reflected by the elevated level of malondialdehyde (MDA) [9]. Therefore, attenuation of oxidation-induced RPE cell death as well as lens protein and lipid oxidation may delay the progression of atrophic dry type AMD and cataract, respectively. 

Although dry type AMD is a leading cause of blindness, only a limited battery of effective treatments is available for this disease. Similarly, cataract treatment is limited to surgical replacement of the opacified lens, which is not an easily accessible option in developing countries. Therefore, new topical drugs that will attenuate AMD and cataract development are needed. A common causal factor for both diseases is oxidative stress, thus, cellular antioxidant mechanisms may play a major role in their prevention. Indeed, vitamin E and NAC were shown to significantly mitigate the effect of oxidative stress in human retinal pigment epithelial (RPE) cells (line ARPE-19) [10]. Furthermore, treatment of mice exposed to phototoxic doses of light with *N*-acetyl-cysteine amide (NACA) maintained RPE cell integrity, prevented death of outer nuclear layer cells and rescued photoreceptor function, indicating that NACA protected RPE and photoreceptor cells from oxidative stress-induced cell death [11]. Both NAC and NACA are supplying cells with cysteine, the rate limiting substrate in glutathione biosynthesis, thereby increasing glutathione cellular content that protects them from oxidative stress. Also, the natural antioxidant defense mechanism was shown to prevent molecular photo-damaging effects of solar radiation in lens crystallins in vitro [12]. Several antioxidant compounds were shown to attenuate cataract progression: (i) Curcumin, the bioavailability of which is limited [13]; (ii) acetyl-l-carnitine [14]; (iii) NAC in combination with the allicin derivative diallyl disulfide [15]; (iv) Negerloxin [16]; (v) Quercetin and its derivative Rutin [17,18]. 

Antioxidant mechanisms are regulated by the transcription factor nuclear factor erythroid 2-related factor 2 (Nrf2) and include glutathione as the first line and the phase II antioxidants enzymes as the second line of the cellular defense [19]. Allicin, the active component of garlic, was shown to elevate the activity of Nrf2 and thereby to enhance the GSH/GSSG ratio and modulate the level of ROS-associated enzymes [2]. These data suggest that allicin may exert a protective effect against H_2_O_2_-induced cytotoxicity in RPE cells via ROS regulation. 

*S*-Allylmercapto-*N*-acetylcysteine (ASSNAC) is a conjugate of NAC with the active residue of allicin, *S*-allylmercaptan, rendering the molecule a hydrophobic nature that improves its cell and tissue permeability. We have shown in vascular endothelial cells that ASSNAC interacts with free cysteine residues on Keap1, the oxidative stress sensor of Nrf2, probably via thiol-exchange reaction. This process results in the release of free NAC from ASSNAC and the release of Nrf2 from the Keap1-Nrf2 complex followed by Nrf2 nuclear translocation and activation [20]. This ASSNAC-induced Nrf2 activation increased the expression of phase II detoxifying enzymes, including the cystine transporter (xCT), which provides cells with free cysteine as a substrate for glutathione biosynthesis. Thus, ASSNAC dual anti-oxidant activity includes increased cellular availability of cysteine (via released NAC and up-regulated cysteine transporter activity) and up-regulated phase II-induced enzymes for glutathione biosynthesis, both leading to significant increase in glutathione cellular level, resulting in significant augmentation of cells [20] and of the entire organism [21] resistance to oxidative stress. Indeed, our further study demonstrated a protective role for ASSNAC against oxidative stress, as it attenuated the clinical symptoms in experimental autoimmune encephalomyelitis (EAE), which serves as a mouse model for multiple sclerosis [22]. Therefore, in the present study we further assessed ASSNAC ability to prevent oxidative stress-induced ARPE-19 cell death in vitro and lens opacification in an ex-vivo lens model.

## 2. Materials and Methods 

### 2.1. Materials

ASSNAC was synthesized as previously described [20]. Briefly, cold sodium thiosulfate and allyl bromide were mixed to form allylthiosulfate, which was further mixed with a cold solution of NAC (at pH 8.0) to form ASSNAC. This ASSNAC was extracted at room temperature (at pH 3.0) by *t*-Buthyl methyl ether (MTBE) that was evaporated under vacuum conditions and the resulting ASSNAC, in the form of a yellowish oil, crystallized. The purity of the preparation was assessed by HPLC using a C:18 hydrophobic column and the ASSNAC appeared as a major peak representing 96.8% of the loaded material at a retention time of 6.23 min. One contaminating peak of a less hydrophobic material at a retention time of 3.91 min (which differs from NAC retention time) representing 3.2% of the loaded material was also detected. Preparation of an ASSNAC aqueous stock solution: 100 mg ASSNAC is dissolved in 8.48 mL DDW plus 2.12 mL Na_3_PO_4_ (0.2 M) resulting in ASSNAC final concentration of 40 mM in phosphate buffer (40 mM; pH = 7.4) and further diluted in PBS and kept at 4 °C. 

Chemicals: NAC, 5-Sulfosalicylic acid (SSA), GSSG, 2-vinylpyridine, 5,5’-dithiobis(2-nitrobenzoic acid) (DTNB), β-nicotinamide adenine dinucleotide 2’-phosphate reduced tetrasodium salt (NADPH), glutathione reductase, bovine serum albumin (BSA), neutral red dye, homocysteine and tert-butyl hydroperoxide (*t*-BuOOH) were from Sigma (St. Louis, MO, USA). H_2_O_2_ was obtained from Merck (Kenilworth, NJ, USA). Dulbecco’s modified eagle’s medium (low glucose) (DMEM), F-12 medium, antibiotics, glutamine and fetal calf serum were from Biological Industries (Beit Haemek, Israel). Tissue culture dishes were from Thermo Fisher Scientific Nunc (Roskilde, Denmark). Monobromobimane (mBBr) was obtained from Molecular Probes, an Invitrogen company (Eugene, OR, USA, http://www.invitrogen.com).

### 2.2. Cells and Tissue Culture Conditions

The ARPE-19 human RPE cell line was purchased from the American Type Culture Collection (Manassas, VA, USA). ARPE-19 cells are an accepted cell model for the study of oxidative stress and anti-oxidants effects on RPE cells [23,24,25,26]. Cells were cultured in DMEM/F-12 (1:1) supplemented with 10% heat-inactivated Fetal Calf Serum, 2 mM l-Glutamine, 100 U/ml Penicillin, 100 μg/mL Streptomycin and 50 μg/mL Neomycin and passages 3 to 7 were used. ARPE-19 cultures were grown in 35 mm plates for 6 days until confluence, washed and incubated for 24 h in the absence or presence of ASSNAC or NAC in fresh growth medium containing 1% Fetal Calf Serum. Cultures were then washed, lysed and subjected to glutathione and free sulfhydryls determination. For cell survival assays, cells were grown in 24-well plates for 3 days until confluence and then pretreated with or without ASSNAC for 24 h, washed and further exposed to fresh growth medium containing 1% Fetal Calf Serum with or without ASSNAC and exposed to *t*-BuOOH, H_2_O_2_ or homocysteine and cell survival was determined. It should be pointed that our previous studies with cultured vascular endothelial cells [20] and nerve cells [22] demonstrated that Fetal Calf Serum does not interfere or quench the activity of both ASSNAC and NAC. On the contrary, the media supplemented with serum protected the cells and reduced the variability between replicate cultures. 

### 2.3. Porcine Lenses Ex-Vivo Model

Porcine eyes (from both males and females) purchased from Lahav C.R.O. (Kibbutz Lahav, Israel) were extracted immediately following slaughter and kept at 4 °C. Within the first 24 h following extraction, eyes were incubated at 4 °C in DMEM supplemented with a high concentration of antibiotics (1000 U/mL Penicillin, 1000 μg/mL Streptomycin and 125 U/mL Nystatin) for 3 h. 

Lenses were surgically isolated under sterile conditions, inspected under a binocular (4×) and intact lenses were cultured in the absence or presence of 0.2 or 1.0 mM ASSNAC for 24 h at 37 °C under 10% CO_2_ and atmospheric oxygen. The culture medium consisted of low glucose DMEM, as recently described [27], supplemented with 2 mM l-Glutamine, Non-essential amino acids, 100 U/mL Penicillin, 100 μg/mL Streptomycin and 12.5 U/mL Nystatin. 

A second group of lenses was further incubated for additional 24 h in the absence or presence of 0.5 mM H_2_O_2_. Media from both groups were collected for glutathione determination. Opacity (optical density (O.D.)) of lenses from both groups was determined and then lens capsules (adhering epithelial cells) and cores (fiber cells consisting the lens cortex and nucleus) were isolated on a cold plate under a binocular (4×) and subjected to glutathione and free sulfhydryls determination. 

### 2.4. Glutathione Determination

The samples for glutathione determination were prepared as follows: (i) ARPE-19 cultures were washed with PBS, lysed in 10 mM HCl by three cycles of liquid nitrogen freezing and thawing. Proteins were precipitated by addition of SSA (10%) followed by centrifugation (10,000 g) and supernatants were collected as we have previously described [20]; (ii) Lens capsule and core samples were placed in cold HCl (10 mM) solution, homogenized using Polytron (Kinematica, Luzern, Switzerland) for 1 min followed by three cycles of liquid nitrogen freezing and thawing as we have previously described for mouse brain and spinal cord tissue samples [22]. Proteins were precipitated as described above and supernatants collected; (iii) lens incubation medium was collected and HCl was added to a final concentration of 10 mM. 

Glutathione (reduced (GSH) and oxidized (GSSG)) was determined as we have previously described [20,22] by the Anderson recycling method [28], which was shown to be accurate, well-established and widely used [29]. To determine GSSG, 2-vinylpyridine was used to conjugate GSH and remove it from the mixture as described [28]. In lens samples, glutathione values are presented as nmole/lens. In ARPE-19 samples, protein pellets were lysed in 0.5 N NaOH and quantified by the Lowry method [30] using BSA as standard and glutathione values are presented as nmole/mg protein.

### 2.5. Free Sulfhydryls Assay

Quantitative determination of non-protein and protein sulfhydryls in cell extracts was performed using mBBr, a cell membrane permeable reagent that alkylates free sulfhydryls according to Kosower and Kosower [31]. Cultures were incubated in DMEM without phenol red and treated with mBBr (0.1 mM; 5 min) as described [20], exposed to lysis buffer (1.0 mL; 20 mM Tris-HCl, pH 7.4, 150 mM NaCl, 1% Triton X-100, 2.5 mM EDTA and 2.5 mM EGTA) for 30 min at room temperature and centrifuged. An aliquot (0.2 mL) of the Triton-soluble supernatant, representing the cellular cytosol, was taken to determine the total free sulfhydryls by measuring the amount of fluorescence with a FLx800 fluorimeter (BioTek Instruments, Winooski, VT, USA) as previously described [31] (excitation at 380 nm, emission at 480 nm), using mBBr-labeled GSH as a standard. Triton-soluble proteins were precipitated with 10% TCA followed by centrifugation and an aliquot (0.2 mL) of the supernatant was taken for measurement of fluorescence representing the non-protein free sulfhydryls. Treatment with TCA reduced the fluorescence values by a factor of 1.6, consequently, the level of non-protein free sulfhydryls was corrected accordingly. The amount of protein free sulfhydryls was calculated from the difference between the total free sulfhydryls and the non-protein free sulfhydryls. Data are presented as nmole/dish.

### 2.6. Cell Survival Assay

Confluent cultures grown in 24-well plates were treated with various reagents and cell survival was determined by the neutral red staining method [32]. Briefly, cells were incubated in 1% neutral red solution in DCCM-1 for 2 h at 37 °C, washed and the dye content of the cells representing cell survival was extracted with the Sorrenson solution and determined at 550 nm.

### 2.7. Lens Opacity Measurement

Lens opacity was measured by a photometric evaluation of lens absorbance as recently described by Bree and Borchman [33]. This recent study compared spectroscopic measurement versus grading of lenses from photographs and concluded that lens absorbance measurements have an advantage over grading of lenses from photographs because photographic grading only quantifies opacity into 4 to 5 grades whereas spectroscopic measurement provides a continuous grading and is not subjective. As described by Bree and Borchman [33], the porcine lenses were easily placed in a spectrophotometer polystyrene disposable cuvette (10 × 10 × 45 mm–3 mL cuvette; Sarstedt, Nuembrecht, Germany) containing PBS. The cuvette was positioned in a spectrophotometer (Shimadzu Corp., Tokyo, Japan) with the light source stationed perpendicular to the lens and the light beam crossing the center of the lens. The absorbance spectrum, between 400 to 700 nm, of a fresh lens was monitored and stable absorbance was observed at a wave length of about 500 nm. This wave length was further used in all lens opacity measurements. This quantitative method is suggested to give more accurate results than the previously used transparency scoring [34] or analysis of lens images [35]. 

### 2.8. Statistical Analysis

**Figure 1:**
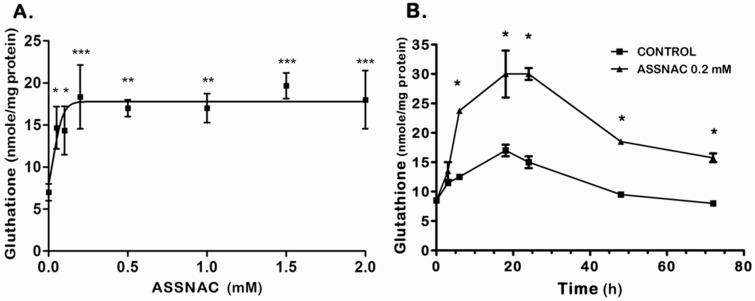
Effect of *S*-Allylmercapto-*N*-acetylcysteine (ASSNAC) on glutathione level in human retinal pigment epithelial (RPE) cells (line ARPE-19)—concentration and time titration. Confluent ARPE-19 cells were incubated in medium containing: (**A**) increasing concentrations of ASSNAC for 24 h, or (**B**) in the absence (control) or presence of ASSNAC (0.2 mM) for the indicated time periods and then glutathione was extracted and determined. Significant differences were marked: * *p* ≤ 0.05; ** *p* ≤ 0.01: and *** *p* ≤ 0.001.

**Figure 2:**
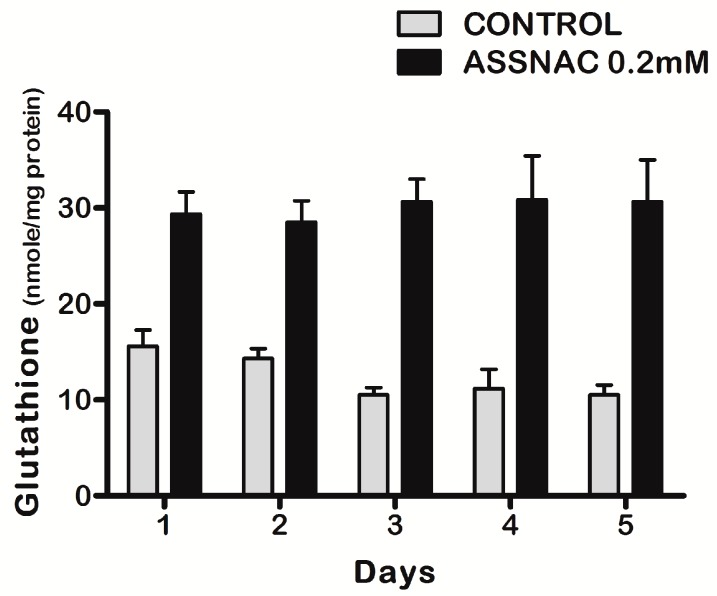
Effect of consecutive daily treatments of ASSNAC on glutathione level in ARPE-19 cells. Confluent ARPE-19 cells were incubated without (control) or with ASSNAC (0.2 mM) consecutively added for 5 days. Cultures were harvested every day and glutathione was extracted and determined. Significant differences between the treated and control groups were found (*p* ≤ 0.01).

**Figure 3:**
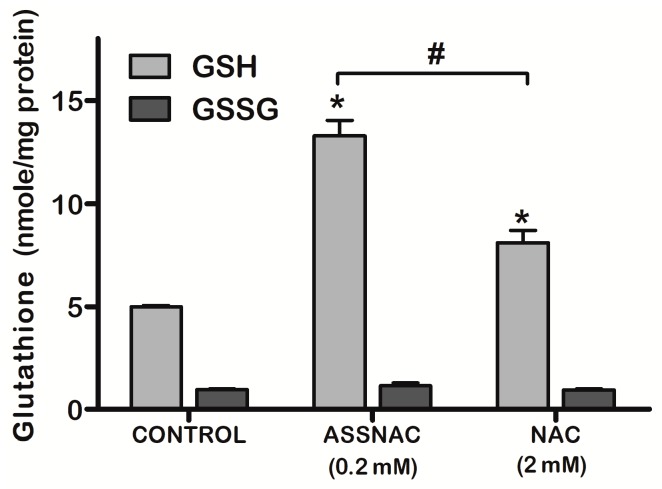
Effect of ASSNAC and *N*-acetyl-cysteine (NAC) on glutathione (reduced (GSH) and oxidized (GSSG)) level in ARPE-19 cells. Confluent ARPE-19 cells were incubated without (control) or with ASSNAC (0.2 mM) or NAC (2.0 mM) for 24 h. Glutathione was extracted and GSH and GSSG cellular contents were determined. GSH content in the ASSNAC and NAC treated groups was significantly higher than in the control (* *p* ≤ 0.01) and significantly higher in ASSNAC than NAC treated group (# *p* ≤ 0.01).

**Figure 4:**
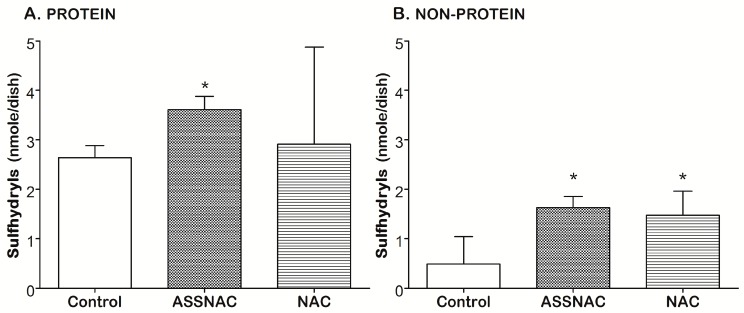
Effect of ASSNAC and NAC on free sulfhydryls level in ARPE-19 cells. Confluent ARPE-19 cells were incubated without (control) or with ASSNAC (0.2 mM) or NAC (2.0 mM) for 24 h. Cells were labeled with mBBr, washed, lysed, and the protein free sulfhydryls (**A**) and non-protein free sulfhydryls (**B**) were determined. ASSNAC and NAC significantly increased total and non-protein sulfhydryls, while only ASSNAC significantly increased protein sulfhydryls (* *p* ≤ 0.05).

**Figure 5:**
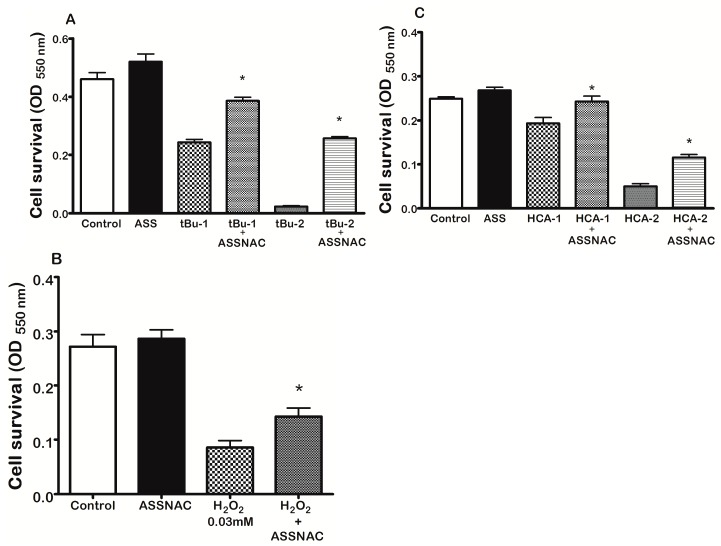
Protective effect of ASSNAC from oxidative stress in ARPE-19 cells. Confluent ARPE-19 cells were pretreated without or with ASSNAC (0.2 mM; ASS) for 24 h, washed and further incubated in medium containing 1% serum without (control) or with ASSNAC (0.2 mM), respectively and exposed to the following oxidants: (**A**) tert-butyl hydroperoxide (*t*-BuOOH) (*t*Bu-1 at 0.05 mM and *t*Bu-2 at 0.10 mM) for 24 h. (**B**) H_2_O_2_ (0.03 mM) for 1 h. (**C**) Homocyesteine (HCA-1 at 7.5 mM and HCA-2 at 10.0 mM) for 24 h. Cultures were washed and stained with neutral red to determine cell survival. Survival of the ASSNAC-treated groups was significantly higher than that of the non-treated group (* *p* ≤ 0.01).

**Figure 6:**
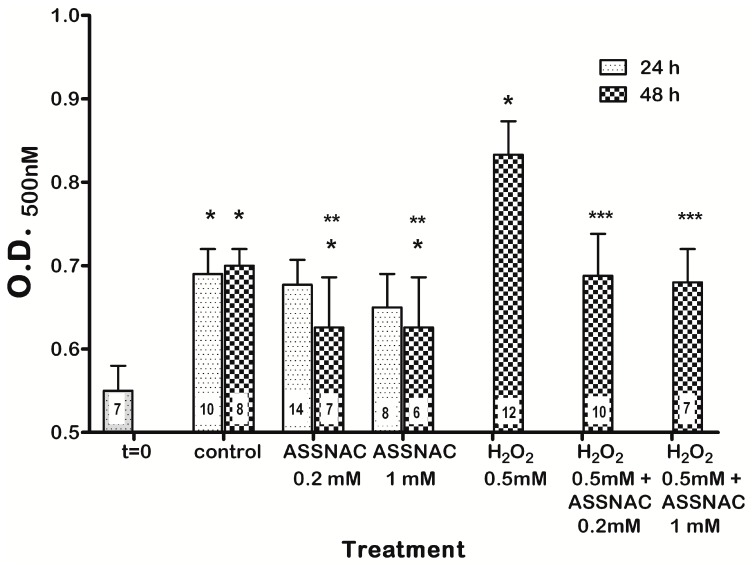
Effect of ASSNAC on lens opacity in an ex-vivo lens cataract model. Lenses opacity was determined on fresh lenses (*t* = 0) and on lenses cultured without (control) or with ASSNAC (0.2 or 1.0 mM) for 24 h. In parallel, opacity of control and ASSNAC-treated lenses that were incubated for additional 24 h without or with H_2_O_2_ (0.5 mM) was determined. The number of lenses per each treatment is indicated in each bar. Significant differences were marked: * *p* ≤ 0.05 versus *t* = 0; ** *p* < 0.05 versus control group; *** *p* < 0.05 versus H_2_O_2_ group.

**Figure 7:**
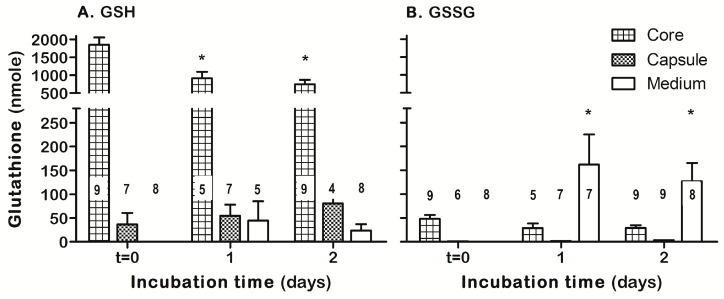
Time-dependent glutathione content in lens core and capsule. Fresh lenses (*t* = 0) or those cultured for 1 or 2 days were collected for glutathione determination. Media were harvested and lenses core and capsule were separated. The level of GSH (**A**) and GSSG (**B**) in the core, capsule and medium were determined. The number of lenses per each treatment is indicated in each bar. Significant differences were marked: * *p* < 0.05 versus *t* = 0).

**Figure 8:**
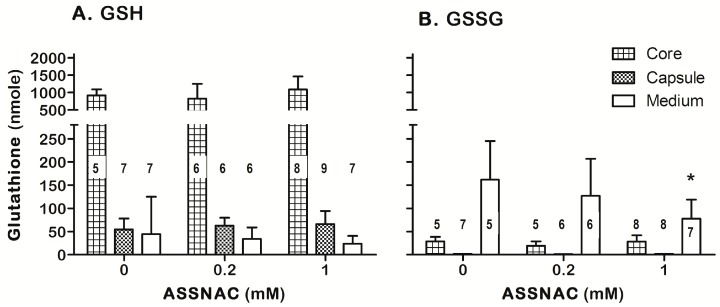
Effect of ASSNAC on GSH and GSSG content in lens core, capsule and incubation medium. Lenses were cultured without (0; control) or with ASSNAC (0.2 mM or 1 mM) for 1 day. Media were harvested and lens core and capsule were separated. The level of GSH (**A**) and GSSG (**B**) were determined. The number of lenses per each treatment is indicated in each bar. Significant difference was marked: * *p* < 0.05 versus control.

The experiments with the ARPE-19 cells were performed 3 times in duplicates (Figures 1 and 2) or once in triplicates (Figure 3) or duplicates (Figure 4) or tetraplicates (Figure 5). The experiments with lenses were performed with 6 to 14 (Figure 6) or 4 to 10 (Figure 7) or 5 to 9 (Figure 8) lenses per each treatment. The results are presented as mean ± SD. The significance of the results was tested using the following tests: 1-way ANOVA Newman-Keuls Multiple Comparison Test (Figures 1A, 4 and 5), 2-way ANOVA (Figures 1B and 2) and 2-way ANOVA Bonferroni posttests (Figure 3). Results of experiments with lenses are presented as mean ± SD and the significance was tested by student’s *t*-test (Figures 6–8). Differences of *p* ≤ 0.05 were considered significant. 

## 3. Results

### 3.1. ASSNAC Up-Regulates Glutathione and Total Free Sulfhydryls Level in ARPE-19 Cells

Exposure of ARPE-19 cultures to increasing concentrations of ASSNAC for 24 h demonstrated a concentration-dependent increase in total cellular glutathione, reaching a maximal effect (2.5-fold versus control) at 0.2 mM ASSNAC followed by a plateau up to 2 mM (Figure 1A). Treatment with ASSNAC (0.2 mM) for various time periods demonstrated a time-dependent increase in total glutathione, reaching a maximal response after about 24 h followed by a slow decline in both control and treated cultures, however, still maintaining a 2-fold increase in ASSNAC-induced glutathione after 72 h (Figure 1B). Consecutive daily treatments of ARPE-19 cultures with ASSNAC for up to 5 days resulted in a sustained 2- to 3-fold increase in total glutathione (Figure 2).

The effect of ASSNAC on glutathione level was compared to that of NAC (Figure 3). In our previous study [20], the optimal NAC concentration was 5- to 10-fold higher than that of ASSNAC. Therefore, cultures were treated with 2.0 mM NAC and demonstrated partial increase in total glutathione which was about 60% of that observed with ASSNAC. The increase in total glutathione following both NAC and ASSNAC treatment was the result of an increase in GSH (about 2.6-fold) with no change in the level of GSSG. The GSH level in ASSNAC treated cultures was significantly higher compared to NAC treated cultures (Figure 3).

Analysis of the cellular content of free sulfhydryls in control ARPE-19 cells demonstrated about 85% of the sulfhydryls to be in the protein cellular fraction (Figure 4). ASSNAC and NAC treatment resulted in a significant increase in total sulfhydryls (190% and 162%) and non-protein sulfhydryls (320% and 300%), respectively (Figure 4B). However, only ASSNAC significantly increased the protein sulfhydryls fraction (140%; Figure 4A).

### 3.2. ASSNAC Protects ARPE-19 Cells from Oxidation-Induced Cytotoxic Effect

Survival of ARPE-19 cells pre-treated without or with ASSNAC (0.2 mM; 24 h) and further exposed to 0.05 mM *t*-BuOOH (24 h) was 53% and 83%, to 0.1 mM *t*-BuOOH (24 h) was 6% and 57% (Figure 5A); to 0.3 mM H_2_O_2_ (1 h) was 32% and 53% (Figure 5B); to 7.5 mM homocysteine was 77% and 98% and to 10 mM homocysteine (24 h) was 20% and 47% (Figure 5C), respectively. ASSNAC treatment alone did not affect cell survival. These results demonstrate the significant protective effect of ASSNAC against various oxidants induced cell death. 

### 3.3. ASSNAC Ex-Vivo Effect on Lens Opacity 

Cultured lenses (untreated control) demonstrated a significant spontaneous increase in opacity within the first 24 h compared to *t* = 0, without any further increase within the next 24 h (Figure 6). Treatment of lenses with ASSNAC at both concentrations (0.2 and 1.0 mM) for 48 h but not 24 h, resulted in a significantly lower opacity compared to control, albeit, still higher than that determined at *t* = 0, suggesting partial prevention of lens opacity by ASSNAC. Exposure of lenses to H_2_O_2_ resulted in a significant increase in lens opacity that was completely prevented by 24 h pre-treatment with ASSNAC (0.2 and 1.0 mM) (Figure 6).

### 3.4. ASSNAC Ex-Vivo Effect on Lens Glutathione 

The core (lens cortex and nucleus) and capsule (including the epithelium cellular layer) of either fresh lenses (*t* = 0) or those cultured for 1 or 2 days were subjected to GSH and GSSG determination (Figure 7). The fresh lens core, representing most of the lens mass, contained most of the lens GSH with a low GSSG level (3–5% of total glutathione). Ex-vivo incubation of lenses for 1 day resulted in a significant decrease of about 50% of GSH core content without concomitant increase in core GSSG level but with concomitant secretion of GSSG into the incubation medium. No further significant changes in GSH and GSSG content were observed on day 2 of culture. The lens capsule GSH was increased along the 2 days of incubation, while the GSSG level in the capsule remained negligible (Figure 7). Ex-vivo incubation of lenses with ASSNAC (0.2 and 1.0 mM for 1 day) did not affect the core and capsule GSH content compared to control, however, 1.0 mM ASSNAC significantly decreased (50%) the level of GSSG in the incubation medium (Figure 8).

## 4. Discussion

Reactive oxygen species (ROS)-induced oxidative stress results in oxidation of various bio-macromolecules, which may eventually lead to cataract and retinal diseases such as AMD. Thus, a balanced redox state is crucial for maintenance of lens opacity and retinal function. A high level GSH with a low level of GSSG is believed to play a key role in this process. Therefore, it is essential to have a functional antioxidant protective system to prevent the development and progression of these diseases. Although glutathione is a major component of this protective system, its cellular biosynthesis is limited due to the shortage in its precursor, cysteine. Therefore, supplementation of cysteine precursor molecules, such as NAC, NACA and 2-Oxothiazolidine-4-carboxylate was shown to increase glutathione level and attenuate oxidation-induced degenerative diseases, including cataract and AMD [11,36,37]. Indeed, NAC was shown to significantly mitigate the effect of oxidative stress in ARPE-19 cells and therefore is suggested to have a potential therapeutic effect for AMD patients [10]. Similarly, NACA was shown to protect RPE cells from oxidative injury [11]. Also, treatment of ocular diseases such as cataract with the antioxidant *N*-acetylcarnosine prevented and partially reversed cataracts within 3–6-months of topical ocular use [38]. Furthermore, treatment with a combination of antioxidants in a Retinitis pigmentosa mouse model increased the glutathione peroxidase activity and glutathione level, while decreasing the number of apoptotic cells in the retina, suggesting a role for retinal thiols and thiol-dependent peroxide metabolism in the survival of photoreceptor cells [39].

Consequently, antioxidant protective compounds continue to merit further investigation. In continuation with this line, we have previously shown that ASSNAC is the only agent with a dual anti-oxidant protective activity (Nrf2 activation and cysteine supplementation) and therefore, it is a highly effective compound reducing oxidative stress in vascular endothelial and nerve cells in culture as well as attenuating clinical symptoms in a multiple sclerosis mouse model [20,22]. In the present study, we further explored the ASSNAC potential protective effect in ocular tissues such as human retinal cells and porcine lenses. 

Indeed, ASSNAC was found to increase the glutathione level in ARPE-19 cells by supplementing the essential cysteine as well as inducing Nrf2 that controls the entire antioxidant machinery. The effect of ASSNAC on ARPE-19 cells was shown by us to be superior to that of NAC as we previously demonstrated in endothelial and nerve cells [20,22], its optimal concentration being 5–10-fold lower and its maximal effect being higher. ASSNAC efficiently protected the cells from acute oxidative stress (short exposure to a high concentration of an oxidant) induced by H_2_O_2_ or chronic oxidative stress (long exposure to a low concentration of an oxidant) induced by *t*-BuOOH or homocysteine. ASSNAC was shown to go through a thiol-exchange reaction with free sulfhydryls but not with oxidants [20]. Therefore, the protective effect of ASSNAC in ARPE-19 cells is not due to a direct interaction and quenching of the oxidants but rather via a cellular protective mechanism similarly to the previously observed effect of other compounds such as 17-β Estradiol, pigmented melanin, and Nrf2 activators [40,41,42]. 

We tested sulfhydryls cellular content in ARPE-19 cells and found that in control cultures 85% of the sulfhydryls are in the protein cellular fraction and 15% in the non-protein fraction, probably representing glutathione, free cysteine, and methionine. ASSNAC was more effective than NAC in increasing GSH. ASSNAC and NAC treatments resulted in a significant increase of about 3-fold in non-protein sulfhydryls that may suggest the capacity of these treatments to probably increase the cellular content of glutathione or its precursor molecules. 

Under ex-vivo incubation conditions, lenses were exposed to atmospheric oxygen [partial oxygen pressure (pO_2_) range between 135 to 157 mmHg] [43], which is significantly higher than pO_2_ at the anterior and posterior surfaces of the lens in the intact eye (in the range of 2.8 to 8.7 mmHg) [44]. This sharp 20- to 40-fold increase in pO_2_ in the ex-vivo incubated lenses probably caused rapid and massive oxidation of relevant components in the lens and may explain the significant increase in lens opacity within the first day of ex-vivo incubation. This observation is in agreement with a previous report on a transient increase in lens opacity in the first days of culture [45]. ASSNAC partially prevented the spontaneous opacity occurring upon exposure of lenses to the atmospheric oxygen but only at a concentration of 1.0 mM. Further exposure of the spontaneously-opacified lenses to H_2_O_2_ for one day, significantly increased lens opacity but this additional opacity was almost completely prevented by pre-treatment with ASSNAC, similarly to the previously reported effect of Sulforaphane [35] and NACA [46]. The ex-vivo treatment with H_2_O_2_ (0.5 mM), representing the actual H_2_O_2_ concentration found in human cataract lenses [47], induced lens opacity formation. Therefore, this ex vivo cataract model may represent the in vivo cataract genesis process and our results may suggest the potential of ASSNAC to attenuate this gradual process of cataract development.

Ex-vivo incubation of lenses resulted in a significant decrease in lens core GSH and increase in capsule GSH. The decrease in lens core GSH is in agreement with a previous study [48] and may represent the lower capacity of the lens core to maintain the GSH level under ex-vivo conditions due to its lower cellular activity. However, the capsule that includes the cellular epithelium layer may better respond to the high pO_2_ by increasing GSH content. Because GSSG, unlike GSH, is secreted [49,50,51], the high pO_2_-induced increase in GSSG formation under the ex-vivo incubation results in GSSG secretion into the incubation medium. Furthermore, the GSH level in the lens is much higher than that released into the medium, while the GSSG level released into the medium is much higher than its level in the lens. This specific release of most lens GSSG (a natural physiological release [49,50,51]) concomitant with very limited GSH release may indicate that the lenses are intact without any spontaneous cellular content release. Exposure of lenses to ASSNAC significantly reduced the amount of secreted GSSG, probably representing an ASSNAC-induced GSSG recycling to GSH in the lens. 

The ASSNAC effect in preventing cataract is similar to the effect of other molecules such as acetyl-l-carnitine and resveratrol that protect the human lens from oxidative stress-induced damage, thereby preventing the formation of age-related cataract [14,52,53,54].

## 5. Conclusions

We found that a lower concentration of ASSNAC compared to NAC is needed to maximally up-regulate glutathione in RPE cells, thereby better protecting them from *t*-BuOOH-, H_2_O_2_- or homocysteine-induced cell death. These results should be further validated in animal models in order to suggest a potential use for ASSNAC as a protective therapy attenuating the progression of retinal degenerative diseases such as AMD. In parallel, ASSNAC attenuated lens opacity developed following ex-vivo exposure to atmospheric oxygen or H_2_O_2_ and significantly reduced the level of GSSG released into the incubation medium. These results, if supported by future in vivo studies, may suggest that ASSNAC protects the lens from oxidative stress and maintains lens transparency, probably by maintaining a high GSH/GSSG ratio.
